# Nanoporous Gold Monolith for High Loading of Unmodified Doxorubicin and Sustained Co-Release of Doxorubicin-Rapamycin

**DOI:** 10.3390/nano11010208

**Published:** 2021-01-15

**Authors:** Jay K. Bhattarai, Dharmendra Neupane, Bishal Nepal, Alexei V. Demchenko, Keith J. Stine

**Affiliations:** Department of Chemistry and Biochemistry, University of Missouri—St. Louis, Saint Louis, MO 63121, USA; jkbxv3@mail.umsl.edu (J.K.B.); dnfzf@mail.umsl.edu (D.N.); bnff8@mail.umsl.edu (B.N.); demchenkoa@umsl.edu (A.V.D.)

**Keywords:** nanoporous gold, implant, doxorubicin, rapamycin, sustained drug release

## Abstract

Nanoparticles (NPs) have been widely explored for delivering doxorubicin (DOX), an anticancer drug, to minimize cardiotoxicity. However, their efficiency is marred by a necessity to chemically modify DOX, NPs, or both and low deposition of the administered NPs on tumors. Therefore, alternative strategies should be developed to improve therapeutic efficacy and decrease toxicity. Here we report the possibility of employing a monolithic nanoporous gold (np-Au) rod as an implant for delivering DOX. The np-Au has very high DOX encapsulation efficiency (>98%) with maximum loading of 93.4 mg cm^−3^ without any chemical modification required of DOX or np-Au. We provide a plausible mechanism for the high loading of DOX in np-Au. The DOX sustained release for 26 days from np-Au in different pH conditions at 37 °C, which was monitored using UV-Vis spectroscopy. Additionally, we encased the DOX-loaded np-Au with rapamycin (RAPA)-trapped poly(D,L-lactide-co-glycolide) (PLGA) to fabricate an np-Au@PLGA/RAPA implant and optimized the combinatorial release of DOX and RAPA. Further exploiting the effect of the protein corona around np-Au and np-Au@PLGA/RAPA showed zero-order release kinetics of DOX. This work proves that the np-Au-based implant has the potential to be used as a DOX carrier of potential use in cancer treatment.

## 1. Introduction

Doxorubicin (DOX), an anthracycline anticancer drug, has been known for its effectiveness in treating a wide variety of cancers, including breast cancer, ovarian cancer, Kaposi’s sarcoma, and lymphomas [1,2,3]. It is a DNA intercalating agent and works primarily by inhibiting the progression of an enzyme called topoisomerase II [4]. However, its use as a cancer drug has been severely constrained due to its inability to distinguish cancer cells from normal cells, resulting in life-threatening side effects [5]. One of the most serious side effects of DOX is congestive heart failure [6]. It has been found that the rate of cytotoxicity depends on the cumulative dose. Therefore, efforts have been made to improve the targeted drug delivery systems (DDSs) to selectively detect and destroy cancer cells using nanostructures as a carrier [7,8,9]. The most notable nanocarrier for DOX is based on liposomes, where DOX is encapsulated inside the liposomes and delivered to cancer cells through the enhanced permeability and retention effect (EPR) [1]. The PEGylated form of liposomes has been approved by the US Food and Drug Administration (FDA) for treating ovarian cancer and Kaposi’s sarcoma after failure of prior systematic chemotherapy [10]. The non-PEGylated form has been approved in Europe and Canada for treating metastatic breast cancer. Although encapsulating DOX inside these liposomes has significantly reduced the cardiotoxicity, many severe side effects of DOX remained, including hand-foot syndrome, anemia, and oral cancer [11,12].

To improve nanocarrier-based DOX delivery, a variety of other organic and inorganic nanoparticles are the subject of current research. However, due to many prerequisites for the nanocarrier to be considered safe and effective, none of the other formulations have been clinically approved for delivering DOX. Besides being biocompatible, the nanocarriers must have prolonged blood circulation to accumulate in the tumor microenvironment [13]. The other important challenges for the advancement of nanocarrier-based DDS are an aggregation of nanocarriers, premature release of drugs in the normal extracellular environment, adsorption of protein on the surface of nanocarriers, clearance from the renal system, capture by the mononuclear phagocytic system (MPS), the fate of nanocarriers after releasing a drug, and toxicity caused by buildups of nanocarriers in visceral organs [14,15,16]. Moreover, it has been found that only 0.7% (median) of the nanoparticles from the administered dose reach a solid tumor via the EPR mechanism [17]. This is a serious problem when trying to translate cancer-targeting nanoparticles from small animal models to humans. For example, it increases cost and complexity to scale up the production of nanoparticles required to administer a high dose and toxicity due to an interaction of >99% of administered nanoparticles with off-target tissues. Therefore, there is a need to address DOX delivery methods from different perspectives.

Implants present great possibilities for effectively treating a variety of localized cancers. It allows both non-site-specific and site-specific sustained release of the drug, which can continuously attack cancer cells while significantly lowering dose-related systemic side effects of the drug. The need for localized therapy of cancer in order to minimize side-effects has been reviewed [18]. In the site-specific implantation, it drastically lowers the toxicity to distant normal cells. It can be easily implanted at the desired location through an implant applicator or image-guided delivery method with minimal incision. The usage of an implant also helps to improve patient compliance by reducing the frequency of dosing. Varieties of biocompatible materials are explored for designing suitable implants and are generally chosen based on either chemical inertness (e.g., silicone) or biodegradable properties (e.g., PLGA) [19]. These materials have their strengths and weaknesses in terms of physical robustness, chemical inertness, drug loading, release kinetics, and biocompatibility. Diverse types of implants are clinically approved for treating varieties of cancers, most notably for prostate cancer and brain tumors. Prostate cancer can be treated with a high dose of radiation by implanting radioactive seeds (also called brachytherapy) directly in the microenvironment of the prostate tumor [20]. On the other hand, brain tumors can be effectively treated by implanting a carmustine-containing wafer in the brain after surgical removal of a tumor [21]. However, studies for the sustained release of DOX from implants for effective treatment of cancers are limited, mainly because of the drug resistance by the cancer cells. It has been reported that when DOX is given in combination with the rapamycin (RAPA), an immunosuppressive drug, its effectiveness in treating cancer significantly increases [22,23,24]. There is a growing interest in developing implants that are made of porous nanostructured materials [25,26,27]. Examples of materials under development as non-eroding implants include nanoporous anodic alumina, titania nanotubes, and mesoporous silica. The use of these materials provides a means of avoiding burst release associated with biodegradable implant polymers and these materials are also selected as biocompatible, stable, inert, and of high surface area. They do not swell or disintegrate, which is sometimes a desirable property. The control of material properties can be used to obtain sustained release and to alter the release profile by adjusting pore dimensions or by surface modification. For a number of these materials, external stimuli such as magnetic fields or ultrasound can be used to modulate drug release. Applications to orthopedics, coronary stents, and release of cancer drugs are all under active investigation. As emphasized by Seker and coworkers [28,29,30] and by Shih and coworkers [31], nanoporous gold is also of interest as a material for drug release applications. The biomedical and bioanalytical applications have also been reviewed by these authors [32].

**Scheme 1:**
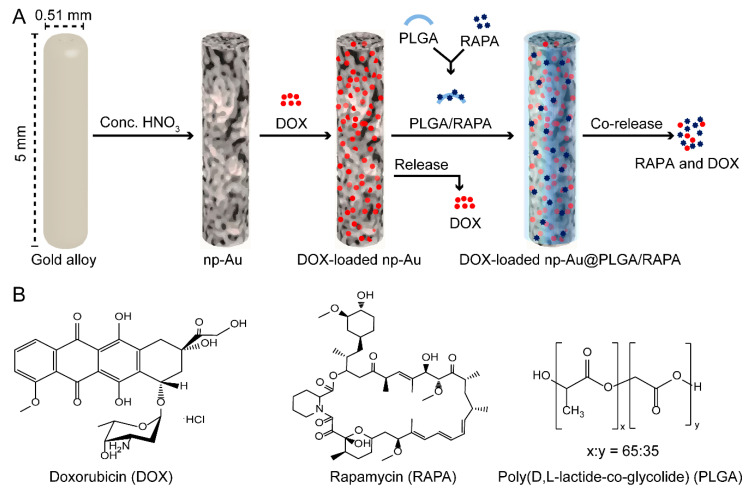
(**A**) Illustration of the fabrication of DOX-loaded np-Au@PLGA/RAPA implant and sustained release of drugs from as-prepared system and (**B**) the chemical structures of DOX, RAPA, and PLGA.

Herein, we demonstrated that a nanoporous gold (np-Au) millirod coated with RAPA containing PLGA (np-Au@PLGA/RAPA) is a promising implant for a sustained, controlled, and parallel release of DOX and RAPA (Scheme 1). The np-Au millirod is a monolithic 3D nanostructure of gold having bicontinuous pores and ligaments with a remarkably high surface area to volume ratio [33]. Furthermore, the np-Au is biocompatible and chemically inert with tunable pores sizes and volumes [34]. Seker’s lab has previously envisioned the possibility of using np-Au as DDS studying the effect of the ionic environment on the release of surface-immobilized fluorescein [28,29]. Here we show that np-Au has excellent DOX encapsulation efficiency without any chemical modification of DOX or np-Au. We have also found that the release kinetics of DOX from np-Au is pH dependent at 37 °C. On the other hand, PLGA is a biocompatible and biodegradable material approved by the FDA as a drug carrier [35]. It degrades by hydrolysis into glycolic acid and lactic acid releasing trapped RAPA. The goal of this research is not to test whether the released drugs kill cancer cells in animal models, but to explore interfaces and interactions of np-Au with unmodified DOX and how np-Au controls the release mechanism. Therefore, we did not perform any experiment using animal models or cell lines but focused on examining the in vitro release kinetics. However, gold and its nanostructures are known for their biocompatible nature [36,37,38]. Gold coating of medical implants [39] has become a fairly common practice. Nanoporous gold electrodes are being developed for neural stimulation [40,41,42,43]. Our results demonstrate that np-Au@PLGA/RAPA is a promising candidate for the co-release of DOX and RAPA, which can have promising applications in cancer treatment.

## 2. Materials and Methods

### 2.1. Materials and Reagents

White gold round wire (10K, 24 GA) was purchased from Hoover and Strong (Richmond, VA, USA). It is an alloy of 41.8% gold and 58.2% of proprietary ratios of nickel, copper, and zinc. Poly(D, L-lactide-co-glycolide) (PLGA, lactide:glycolide = 65:35, MW = 40,000–75,000) and rapamycin (RAPA, ≥95%) were purchased from Sigma–Aldrich (St. Louis, MO, USA). Doxorubicin hydrochloride (DOX) was purchased from TCI America and newborn calf serum was purchased from ThermoFisher Scientific. Antibiotics (Penicillin/streptomycin), solvents (HPLC grade), and buffers were obtained from Sigma-Aldrich. Ultrapure water (18.2 MΩ.cm at 25 °C) was prepared using a Simplicity UV system from Millipore Corporation.

### 2.2. Preparation of np-Au Millirods

The np-Au millirods were prepared by submerging 5 mm long gold alloy wires (diameter = 0.51 mm) in concentrated HNO_3_ for 24 h at room temperature [44]. This process selectively removes other reactive metals from alloy creating a bicontinuous network of pores and ligaments of gold. As-prepared np-Au millirods were thoroughly rinsed with milli-Q water and stored in ethanol until further use.

### 2.3. Surface Area Estimation of np-Au

The surface area of the np-Au millirod was estimated using cyclic voltammetry (CV). The experiment was carried out using PARSTAT 2273 potentiostat (EG&G Princeton Applied Research) in a three-electrode setup with Ag/AgCl (KCl sat.) as a reference electrode and coiled platinum wire (diameter = 0.5 mm) as a counter-electrode. The np-Au millirod was fixed on a gold electrode using 2.0 µL of 0.5 wt % Nafion to be used as working electrode. The potential during CV was cycled between 0.0 and +1.6 V at the scan rate of 5 mV s^−1^ in 0.5 M H_2_SO_4_ at room temperature. The charge under the oxide reduction peak of cyclic voltammogram was used to estimate the surface area of np-Au using the conversion factor of 450 μC cm^−2^ [45].

### 2.4. Surface Morphology and Composition of np-Au

The surface morphologies of np-Au were studied using a scanning electron microscope (SEM 6320F)(JEOL USA, Inc., Peabody, MA, USA) at an acceleration voltage of 8 kV. The ligament’s width and interligament gap of np-Au were computed using analySIS (Soft Imaging System GmbH, Munster, Germany). The purity of np-Au implants after dealloying was determined using an energy-dispersive X-ray (EDX) detector (Oxford Instruments, Abingdon, UK) coupled to SEM.

### 2.5. Loading of DOX Inside np-Au

To study the effect of pH on DOX loading inside np-Au, a low concentration of DOX (25 µM) was prepared in acetate buffer (pH 5.5), milli-Q water, and tris buffer (pH 7.4). A high concentration of DOX (1 mM) was prepared in pH 5.5 acetate buffer because of its higher solubility in acidic conditions. The np-Au implants were submerged in DOX solution in centrifuge or microcentrifuge tubes and left in the dark at room temperature for 24 h with gentle shaking. The wires were washed with fresh buffer after loading. The amount of DOX loaded in np-Au was calculated by subtracting the absorbance peak at 480 nm before and after loading (UV–Vis spectrophotometer, Cary 50) (Figure S1) and fitting with a standard curve (Figure S2). The encapsulation efficiency (*EE*) of DOX was determined using the following equation

$$EE\;(\%)\;=\;\frac{\mathrm{Amount}\;\mathrm{of}\;\mathrm{DOX}\;\mathrm{loaded}\;\mathrm{in}\;\mathrm{np}\text{-}\mathrm{Au}}{\mathrm{Total}\;\mathrm{amount}\;\mathrm{of}\;\mathrm{DOX}}\;\times \;100\%$$

### 2.6. Atomic Force Microscopy (AFM) Imaging

Atomic force microscopy (AFM) images were collected in tapping mode for characterizing the morphology of np-Au before and after DOX loading, and the corresponding height distribution profiles were plotted to determine the changes in height. BioScope Resolve^TM^ AFM (Bruker, Santa Barbara, CA, USA) integrated with NanoScope^®^ software and silicon cantilevers TAP300Al-G (Ted Pella, INC, Redding, CA, USA) with a force constant of 40 N/m were used for imaging. The images were processed using Gwyddion software.

### 2.7. Fourier Transform Infrared (FTIR) Spectroscopy

DOX loaded inside np-Au was extracted using pure dimethyl sulfoxide (DMSO) for checking any structural modification of DOX inside np-Au. Then, the DOX was dried at 40 °C by passing nitrogen gas overnight and FTIR-attenuated total reflection (ATR) spectra were collected over the range 4000–650 cm^−1^ at a resolution of 2 cm^−1^ using NICOLET 6700 spectrometer (Thermo Fisher Scientific, Waltham, MA, USA).

### 2.8. Computations

Energy minimization and molecular orbital energy calculation were carried out using ChemBio3D Ultra.

### 2.9. Coating of DOX-Loaded np-Au with PLGA/RAPA Film

The PLGA and RAPA were separately dissolved in dichloromethane (DCM) and DMSO, respectively, to prepare 10% (*w/w*) solutions. They were then thoroughly mixed to create 0.5% (*w/w*) of RAPA in PLGA. The as-prepared PLGA/RAPA mixture was used for dip coating DOX-loaded np-Au. The DOX-loaded np-Au was submerged and rolled for 15 s in the 100 µL PLGA/RAPA mixture in a microcentrifuge tube followed by drying at 37 °C overnight. The loss of DOX during the 15 s of dip-coating is considered as negligible mainly because it is a quick process, the PLGA is highly viscous, and DOX that might come out of np-Au should be trapped within the PLGA layer. SEM imaging was performed to determine the thickness of the PLGA layer around the np-Au implant.

### 2.10. In Vitro Drugs Release Kinetics

For the study of in vitro release, drug-loaded np-Au implants with or without a coating of PLGA/RAPA were incubated in a capped glass vial containing 5 mL of release media at 37 °C. We used four different types of environments as release media: pH 5.5 (acetate buffer), pH 6.6 (phosphate buffers), pH 7.4 (phosphate buffer), and 10% newborn calf serum containing 1% penicillin/streptomycin. At predetermined time intervals, 500 µL of the supernatant was removed and the same volume of fresh medium was added. The collected supernatant containing DOX and RAPA was quantified using UV–Vis spectrophotometer at 480 nm and 279 nm, respectively (Figures S1 and S2).

## 3. Results and Discussion

### 3.1. Fabrication and Characterization of np-Au Millrods

**Figure 1:**
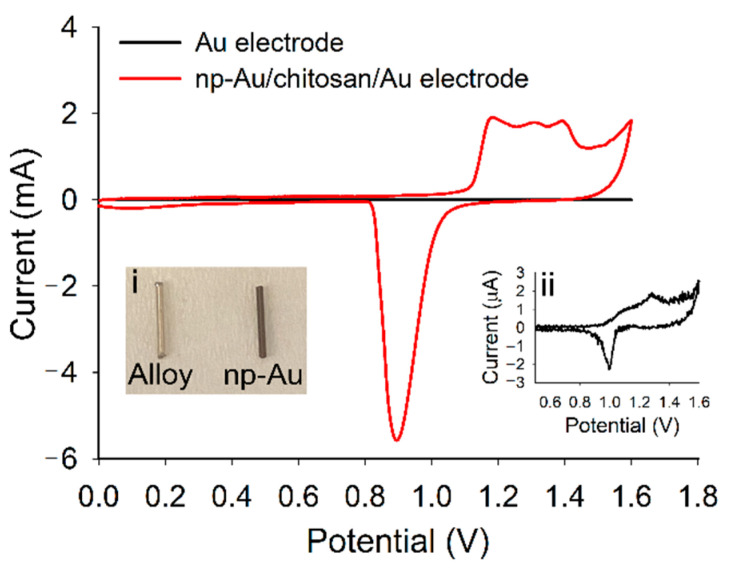
Cyclic voltammogram (CV) of the Au electrode (black) and np-Au millirod placed on the Au electrode (red); insets (i) is a photographic image of alloy and np-Au millirod (length = 5 mm) and (ii) is the magnified CV of the Au electrode.

The np-Au millirods were prepared by leaching the reactive metals from Au alloy millirods by submerging in concentrated HNO_3_ for 24 h. The specific surface area of the as-prepared np-Au was determined by cyclic voltammetry using the gold oxide-stripping method and found to be 6.04 ± 0.54 m^2^ g^−1^ (Figure 1). This value is comparable (6.4 m^2^ g^−1^) to our previous work when determined using the BET surface area analysis [46]. For implants, it is appropriate to express specific surface area as per unit volume rather than mass and was found to be 25.53 m^2^ cm^−3^. This high surface area-to-volume ratio of np-Au millirod is advantageous for loading higher amount of drug molecules. The inset (i) of Figure 1 shows a change in the metallic color of Au alloy to dark color after dealloying indicating the formation of nanopores. The inset (ii) of Figure 1 is the magnified CV of the Au electrode, the surface area of which is negligible compared to the surface area of np-Au.

**Figure 2:**
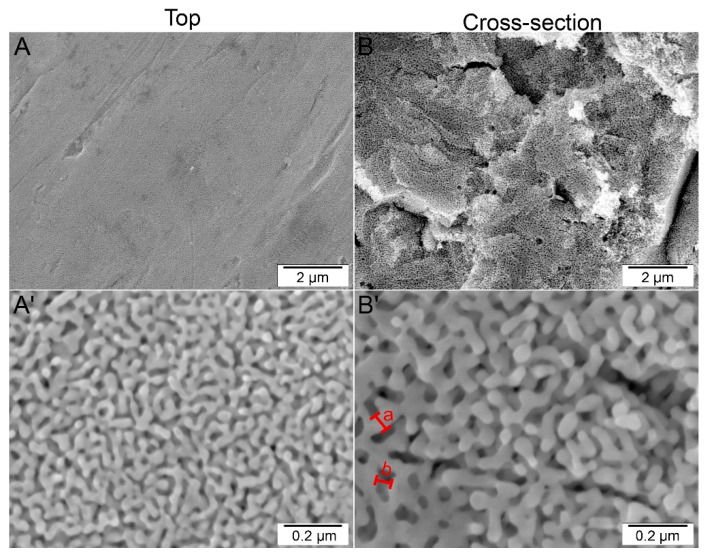
SEM images of top view (**A**,**A’**) and cross-sectional view (**B**,**B’**) of np-Au millirod at low (**A**,**B**) and high (**A’**,**B’**) magnifications. Letters a and b in figure (**B’**) represent typical ligament width and interligament gaps, respectively.

**Figure 3:**
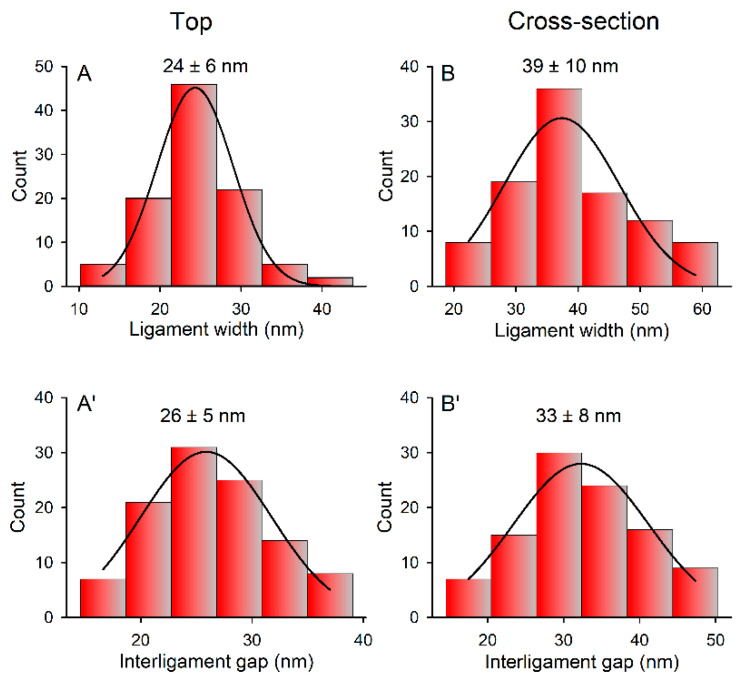
Size distribution of ligament width (**A**,**B**) and interligament gap (**A’**,**B’**) of the top or exterior (**A**,**A’**) and cross-sectional or interior (**B**,**B’**) regions of np-Au.

**Figure 4:**
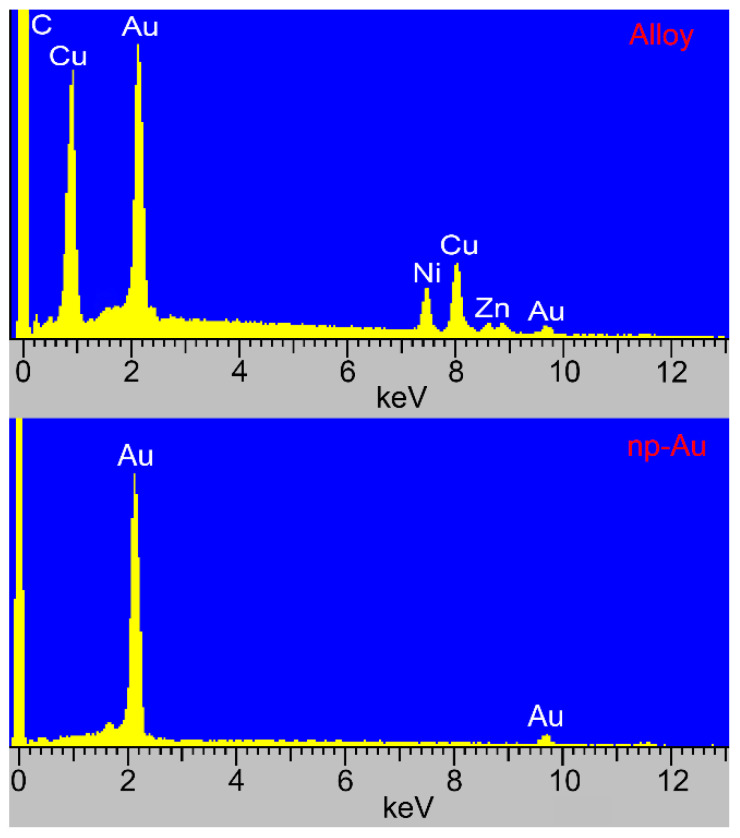
Typical EDX spectra of alloy (**top**) and the np-Au (**bottom**) showing removal of reactive metals present in the alloy after dealloying process.

The surface morphology of the np-Au millirod was characterized using SEM (Figure 2A–B’). We found that the surface morphology varied between the top (exterior) and cross-sectional (interior) regions of np-Au. The exterior surface is crack-free over wide regions with minor scratches on the surface, whereas the interior surface has small domains of structures oriented at different angles due to different crystal planes. The bright regions in the low magnification cross-sectional SEM image are caused by mechanical breakage during sample preparation. However, neighboring darker structures were not affected by the mechanical force due to different crystal orientations. The high magnification SEM images show the characteristic bicontinuous structure of np-Au with ligaments and interligament gaps consistent with previous reports [47]. We found that the interior region has larger ligament widths and interligament gaps compared to the exterior region (Figure 3). The measurements were taken at right angles to the length of ligaments and gaps for the ligament width and interligament gap, respectively. The average ligament width and interligament gap of the exterior region are 24 ± 6 nm and 26 ± 5 nm, respectively, whereas those for the interior region are 39 ± 10 nm and 33 ± 8 nm, respectively. The EDX spectra in Figure 4 illustrate that the np-Au is free from reactive metals present in the alloy.

### 3.2. Loading of DOX at Low Concentration

**Figure 5:**
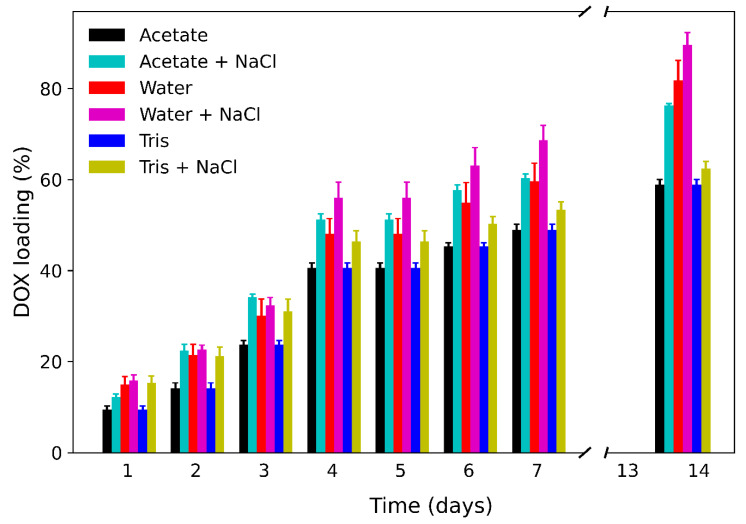
Loading of DOX inside np-Au from low concentration (25 µM) solution at a different pH environment with or without 0.14 M NaCl.

To compare the loading of DOX at a different pH, we prepared an equal concentration of DOX (25 µM) in acetate buffer (pH 5.5), milli-Q water (pH ~ 6–7), and tris buffer (pH 7.4). A low concentration of DOX (25 µM) was used owing to its low solubility at pH 7.4. As shown in Figure 5, it took 4 days for loading 50% of the total DOX loaded, which then loaded slowly until saturation after two weeks. We can also observe that the acidic environment did not have significant advantages in the loading of DOX compared to neutral or slightly basic conditions. This proves that the presence of either the –NH_2_ or –NH_3_^+^ group in DOX did not play a significant role in the loading process. Furthermore, it was found that the presence of 0.14 M NaCl significantly increased the loading capacity under all the experimental pH conditions. This further suggests that electrostatic interactions of NH_3_^+^ with the gold surface are not the dominant factor for determining DOX loading. The pK_a_ value of the primary amine on DOX is reported as 8.2 [48]. The addition of NaCl will diminish unfavorable electrostatic interactions between DOX molecules and hence it is reasonable that at these pH values below the pK_a_ where the amine group is to some extent protonated that the addition of NaCl would favor higher loading over that found in the absence of NaCl if interactions between DOX molecules are the dominant factor. Addition of NaCl also prevented DOX from degrading or changing when acetate buffer was used as a loading buffer (Figure S3). Attempts to use a basic condition (pH 8.4) were unsuccessful because of the low solubility of DOX at a basic pH where the amine group is unprotonated. The loading being the highest in water with NaCl is perhaps due to a lack of interference of buffer ions that may also adsorb on the gold surface to some extent when a buffer is used. The zeta potential of Au was reported on the basis of streaming potential measurements on vacuum sputtered thin gold films to be near −20 mV for the range pH = 6–8 when the pH was adjusted using only KOH or HCl at an ionic strength of 10^−3^ M [49]. The isoelectric point of the Au surface was reported to be at pH = 4.5.

### 3.3. Loading of DOX at High Concentration

**Figure 6:**
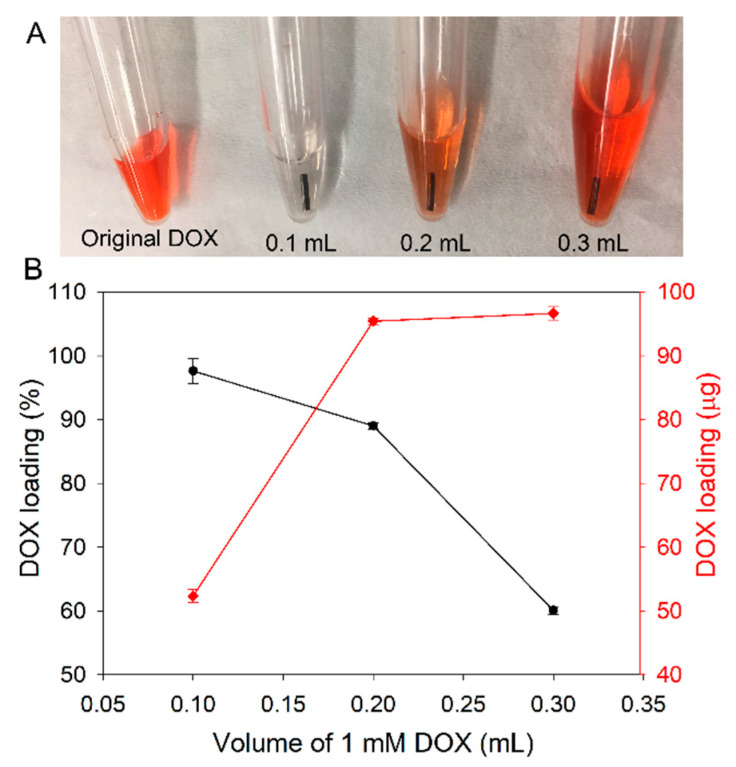
Loading of different volumes of 1 mM DOX on np-Au at pH 5.5. (**A**) Photographic image of original DOX (left) and after 24 h loading on np-Au from 0.1, 0.2, and 0.3 mL DOX solution. (**B**) Amount of DOX loaded on np-Au from a different volume.

To improve the loading rate, a high concentration (1 mM) of DOX was prepared in acetate buffered saline of pH 5.5 owing to its higher solubility in acidic conditions. Attempts to create 2 mM or higher concentrations of DOX in an aqueous solution were not successful. Figure 6A shows an original DOX solution and change in color of different volumes (0.1, 0.2, and 0.3 mL) of 1 mM DOX solution after loading of DOX inside np-Au for 24 h. The reason for monitoring the loading with the change in volume of 1 mM solution is to improve the loading rate while minimizing the waste. As shown in Figure 6B, the loading efficiency of np-Au millirod is close to 100%, when it was incubated in 0.1 mL DOX solution for 24 h. However, maximum loading was obtained from 0.2 mL DOX solution with a loading efficiency of 89%, which is 95.5 ± 0.5 µg per np-Au millirod. Further increasing the volume of DOX to 0.3 mL did not significantly improve the loading efficiency. The loading of DOX in a PLGA millirod of dimensions d = 1.5 mm and L = 8 mm was reported as 2900 µg [50]. If the np-Au millirod of the same size was prepared and loaded as described then 1450 µg of DOX would be incorporated assuming an equivalent outcome. The loading of the np-Au is in the same range of magnitude and could be increased by reducing the pore size. It has been reported that dealloying of a thin alloy film precursor gave nanoporous gold with an average pore size of 28 nm when done at room temperature and that average pore size was reduced to 15 nm by dealloying at 0 °C and to 7 nm by dealloying at −20 °C for the same 4 h period [51]. The reduction of the average pore size by decreasing the temperature of dealloying would provide a means to increase the surface area and hence increase the loading capacity if so desired. The effect of altering pore size on loading capacity and kinetics and on release kinetics remains an area for further study.

**Figure 7:**
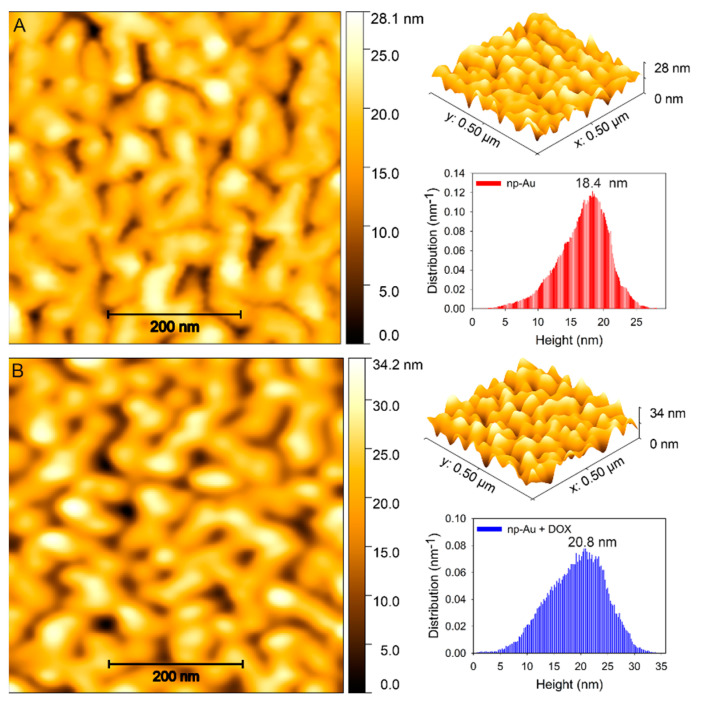
Typical atomic force microscopy (AFM) topographic images of np-Au before (**A**) and after DOX loading (**B**) and their corresponding 3D view and height distribution profiles on the right.

Figure 7 is a comparison of the np-Au surface before and after the loading of DOX using AFM images. The AFM image of DOX loaded np-Au shows an increase in brightness due to increased roughness on the surface caused by immobilized DOX. The height distribution profiles show an increased height of 2.4 nm after loading of DOX on np-Au suggesting the presence of DOX molecules on the np-Au surface, which may be dimerized.

### 3.4. Effect of DMSO on Loading of DOX

**Figure 8:**
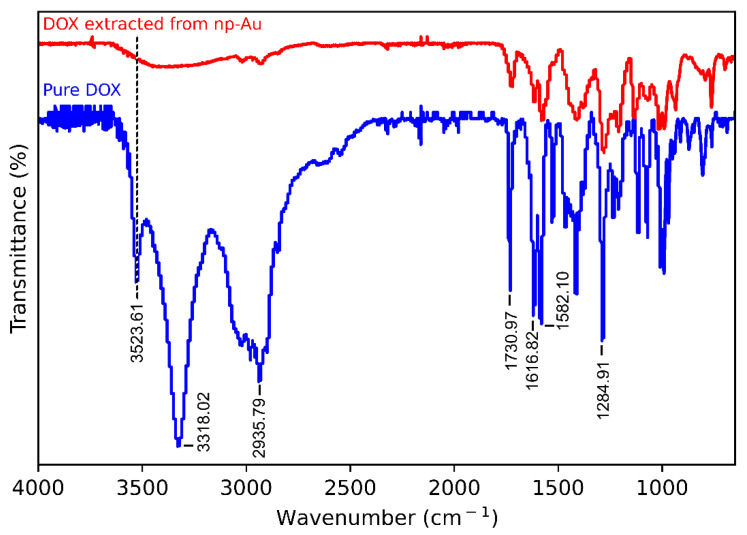
FTIR-ATR spectra of solid samples of pure DOX (blue) and DOX extracted from np-Au by DMSO (red).

DMSO has been frequently used to dissolve and internalize DOX in different nanocarriers [52]. Interestingly, we did not observe any loading of DOX inside the np-Au millirod when DMSO was used as a solvent. DMSO is well-known for accepting protons and breaking hydrogen-bonds [53], which implies that hydrogen bonding is a crucial force for the loading of DOX inside np-Au. DMSO may block the loading of DOX on np-Au by interacting with DOX, np-Au, or both, eventually breaking up hydrogen-bonding. FTIR spectra in Figure 8 correspond to solid samples of pure DOX (blue) and DOX extracted from np-Au four weeks after loading (red). The pure DOX shows characteristic peaks at 3523.61 cm^−1^ (O-H stretch), 3318.02 cm^−1^ (O-H and N-H stretch), and 1731 cm^−1^ (C=O stretch) [54]. Most of the peaks of the extracted DOX were similar to that of the pure DOX except the disappearance of the peak at 3523.61 cm^−1^ and broadening of the peak at 3318.02 cm^−1^. These changes are known to occur due to the complex formation of DOX indicating DMSO directly interacts with DOX.

### 3.5. Mechanism of Loading of DOX on np-Au

In general, the DOX molecules are loaded on the surface of gold nanostructures through intermediate organic molecules by either covalent or electrostatic interactions [55,56]. You et al. reported a very high payload of DOX on citrate-capped hollow gold nanospheres and claimed that it was due to electrostatic interactions between positively charged DOX molecules and negatively charged citrates on the surfaces of nanospheres [57]. However, Curry et al. reported that DOX can directly interact with AuNPs through the Au^+^–π interaction and Au^+^–carbonyl coordination by displacing citrate on the surface of gold nanoparticles [58]. The Au^+^ ions on the surface of AuNPs are believed to be available due to the binding of AuCl_2_^−^ complexes on the surface of AuNPs during synthesis [58,59]. In contrast, there is a low possibility of forming such a complex on the surface of an np-Au millirod, as we employed the top-down fabrication strategy. Nevertheless, we still observed a high loading of DOX in the np-Au millirod. Therefore, it is imperative to understand the forces that help the exceptional loading of DOX inside np-Au.

**Scheme 2:**
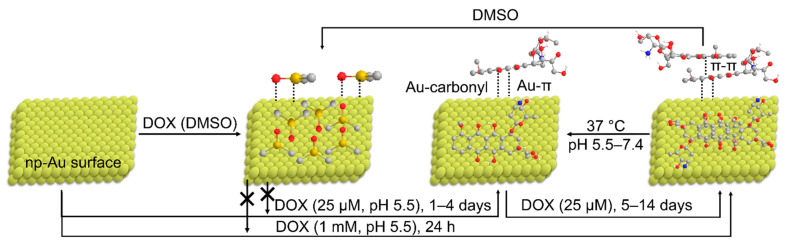
Proposed mechanism of DOX loading on np-Au from high and low concentrations and release conditions.

The proposed mechanism of DOX loading on the np-Au surface and release conditions are illustrated in Scheme 2. As discussed earlier, increasing the ionic strength of the DOX solution does not decrease loading suggesting a lack of ionic interactions between DOX and np-Au. We propose that the excellent loading of DOX on np-Au is due to the combination of different types of interactions between Au atoms and DOX (Au–π and Au–carbonyl) and intermolecular interactions between DOX molecules (π–π and H-bonding). It is well-known that π-conjugated organic molecules readily adsorb on noble metal surfaces minimizing the surface free energy [60]. This should be a driving force for bringing DOX on the surface of np-Au and forming Au-π interactions. It has also been observed that when anthraquinone molecules were brought close to the gold surface, the carbonyl group present on anthraquinone directly interacts with a gold atom [61]. The detailed confirmation of the orientation and interaction of DOX with the Au surface will require the application of advanced spectroscopic methods, such as surface-enhanced Raman (SERS) or surface-enhanced resonance Raman spectroscopy (SERRS), and infrared reflection absorption spectroscopy (IRRAS).

**Figure 9:**
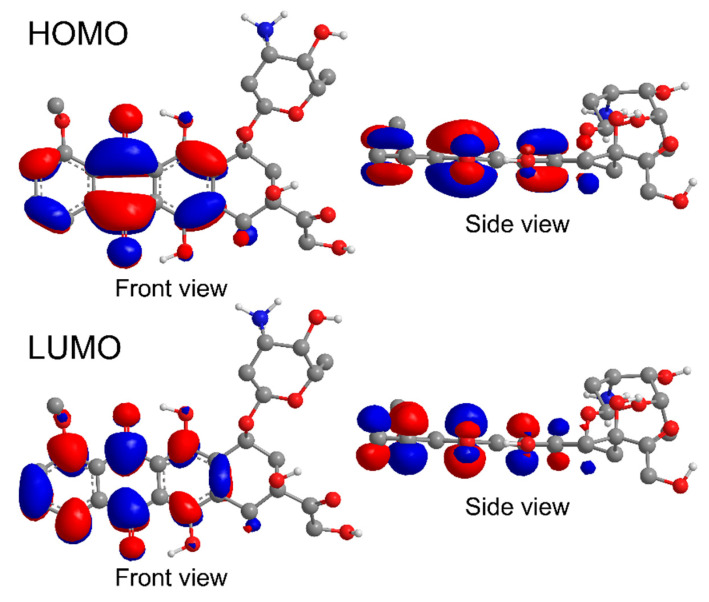
Electrostatic potential surface of HOMO and LUMO of DOX in the front and side view.

Although interactions of sulfur and selenium with gold has been well-known and widely used for creating self-assembled monolayers, the interactions of oxygen, which falls on the same group in the periodic table, with Au has not been well understood. However, many previous works have shown that oxygen can interact with Au when it is attached to a highly electron-rich environment [61,62]. In DOX, the highest occupied molecular orbital (HOMO) is highly localized around the center ring and at the oxygen atoms of the keto group, whereas the lowest unoccupied molecular orbital (LUMO) is nearly equally distributed around anthraquinone moiety (Figure 9). This proves that the Au–carbonyl interaction is highly likely along with the Au–π for loading of DOX on np-Au.

At low DOX concentration (25 µM), we found two distinct loading kinetics at all the three pH conditions, relatively faster loading for the initial 4 days constituting 50% of the total loading and then slower loading kinetics from 5 to 14 days. It is reasonable to say that the first loading step involves only adsorption of DOX on Au, as DOX does not tend to form dimers at low concentration. When a complete monolayer is formed on the np-Au surface, the process of dimerization starts slowly as the unbound DOX approach the surface-bound DOX leading to π–π stacking [63,64]. Finally, an intermolecular network of hydrogen bonding interactions occurs stabilizing the dimer. At high DOX concentration (1 mM), on the other hand, maximum loading was achieved within 24 h supporting the fact that dimerization occurs quickly after the interaction of DOX with Au. However, at a very high concentration, it is also possible that DOX dimerize first in the solution followed by adsorption on the Au surface. To this end, more work needs to be done to better understand the exact mechanism.

### 3.6. PLGA/RAPA Coating

**Figure 10:**
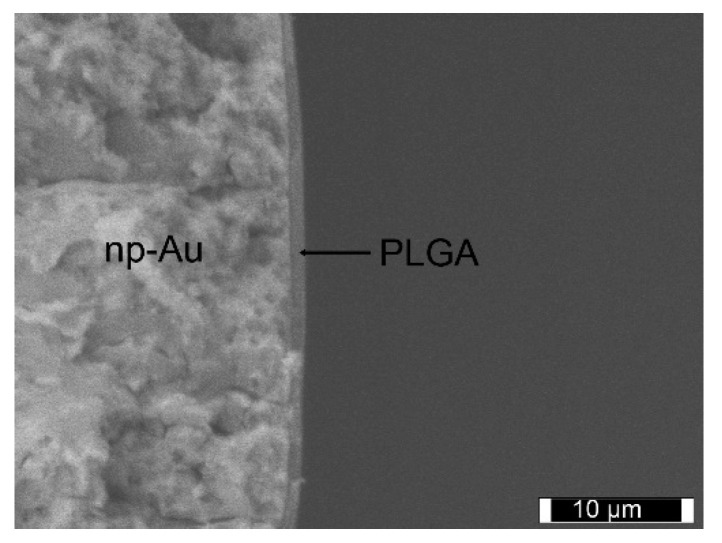
SEM image showing cross-section of np-Au@PLGA/RAPA obtained by single dip coating. The thickness of PLGA coating is ≈1.5 µm. Scale bar:10 µm.

The DOX-loaded np-Au was encapsulated by PLGA containing RAPA using the dip-coating method for combined release of DOX and RAPA. PLGA is well-known for the entrapment of diverse types of therapeutics, and its drug releasing capabilities can be easily tuned by varying its molecular wt., ratio of lactide to glycolide, and size/thickness [65]. We found that single dip-coating creates nearly 1.5 µm thick PLGA coating around np-Au implant, as shown in Figure 10. Multiple dip-coatings can be created by repeating drying and dip-coating steps. However, the release rate of DOX drastically decreased with an increase in thickness.

### 3.7. In Vitro Drug Release

**Figure 11:**
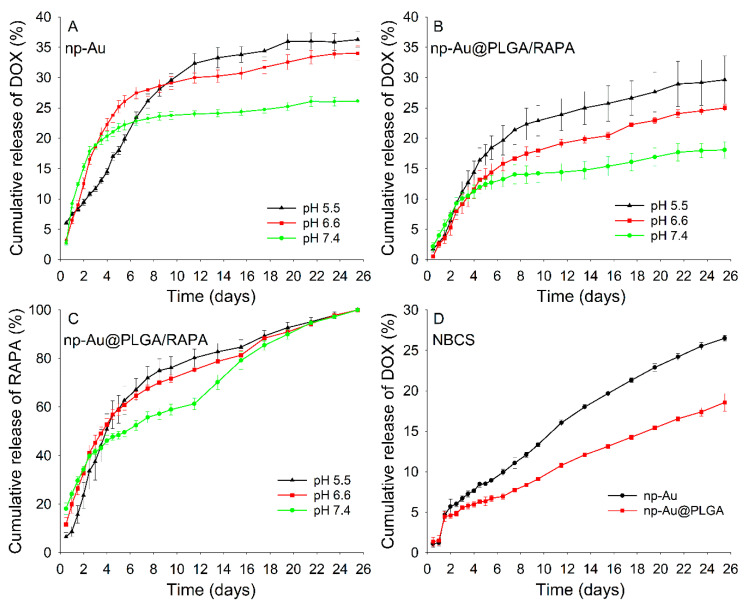
Cumulative release of drugs as a function of time. (**A**) DOX from np-Au in buffers at different pH, (**B**) and (**C**) DOX and RAPA, respectively, from np-Au@PLGA/RAPA in buffers at different pH, and (**D**) DOX from np-Au and np-Au@PLGA/RAPA in newborn calf serum (NBCS) at pH 7.4.

The release behavior of DOX from np-Au and np-Au@PLGA/RAPA was assessed mimicking possible physiological conditions. It was monitored at 37 °C in buffer solutions at three different pH values (5.5, 6.6, and 7.4) and in newborn calf serum at pH 7.4. The release profiles of DOX from np-Au and np-Au@PLGA/RAPA in buffer at different pH are shown in Figure 11A,B, respectively. At all the pH conditions, DOX released from both np-Au and np-Au@PLGA/RAPA showed sustained release for more than 3 weeks. It can also be seen that a higher percentage of DOX released with lower pH conditions. This is important as the extracellular microenvironments of tumor cells is slightly acidic (pH 6.5–6.9) and hence would be advantageous for release of DOX. Most of the DOX released at 37 °C under the experimental pH conditions is due to the disruption of a network of intermolecular hydrogen bonding and π–π stacking releasing the monomeric DOX. This is important as non-monomeric DOX, such as a dimer, cannot effectively interact with DNA the way monomeric DOX can do [10]. Delivering nanoparticles containing high concentrations of DOX directly inside tumor cells always creates the chance of dimerization if burst released, limiting its efficacy.

The release of DOX from np-Au for the first 24 h is a burst release, which comprises 7–9% depending on pH of buffer. The subsequent release of DOX from np-Au shows that the rate is faster at higher pH (7.4 > 6.6 > 5.5) until nearly half of the total amount of drug released is reached. After that, the release rate reverses with increasing pH (7.4 < 6.6 < 5.5), leading to the cumulative release of 26, 34, and 37%, respectively, in 25 days. The former process can be linked to the effect of pH on breaking hydrogen bonding whereas the latter can be associated with increased solubility of DOX at low pH. On the other hand, we found that DMSO can burst release ≈100% of the DOX loaded on np-Au millirod within an hour. This is due to the strong interaction of DMSO with both gold [66] and DOX breaking hydrogen bonds.

Unlike in np-Au, the initial burst release of DOX was not observed for np-Au@PLGA/RAPA. This is due to the PLGA coating over np-Au, which not only efficiently blocks the burst release but also slows down the release rate in a controlled manner. This leads to similar release rates under all the three pH conditions until nearly half of the total amount of drug released is reached. Then the release rates vary significantly under different pH conditions with the low pH condition having a slow release rate.

Although the drug delivery system based only on pure PLGA structures is widely explored, it is still not a perfect system. The major drawback of the PLGA-based delivery system is the high initial burst release [67]. Weinberg and coworkers have shown that the vast majority of DOX loaded inside PLGA implant releases within 24 h with the half-time of ~4 h [50]. In this study, the effect on tumor size was evaluated on day 4 and day 8 after implantation. Using PLGA and np-Au together significantly decreased burst release behavior. The time frame for release of DOX from PLGA depends on the form of PLGA, its molecular weight, and chemical modification of the polymer end groups. The time frames for release studies from PLGA was similar to that followed here for DOX release from np-Au of 26 days. The release of DOX from PLGA microspheres with either acid or ester end groups and molecular weights of 17 or 44 Da was compared over a six-week period [68]. The ester-capped PLGA microspheres showed release with an initial burst phase, followed by a lag phase of several days and an accelerated release phase while the acid-capped PLGA microspheres showed only burst release mostly completed within 7 days. While the release of near 90% was observed from the acid-capped PLGA, release near only 45% was observed for ester-capped PLGA microspheres. DOX conjugated to the end of PLGA by ester linkage and formed into nanoparticles showed a sustained release over a month that was slower for PLGA of molecular weight 10,000 Da than for molecular weight of 5000 Da. In contrast, DOX not conjugated to PLGA burst release within 5 days from DOX/PLGA nanoparticles (d = 200 nm) [69]. The release of DOX from porous PLGA microspheres (d ~ 10 mm) prepared using water-in-oil-in-water emulsions was followed over a 20-day period and nearly 70% was released in combination with paclitaxel [70]. The release of DOX from PLGA microparticles prepared by spray drying was studied and for varied lactide content a burst release was observed with 70% of the drug released within a day. When composite microparticles were prepared with a variable composition of PLGA and poly-L-lactide (PLLA), it was found that 36–48% of DOX was released within the first half hour followed by slower release monitored over a period of 12.5 days [71]. Additional features of the use of PLGA microparticles in drug delivery and release have recently been reviewed [72].

The release of RAPA from np-Au@PLGA/RAPA occurs simultaneously with the DOX, Figure 11C. It starts with the initial burst release, which can be attributed to a quick release of the surface embedded RAPA, followed by sustained release. Although the rate of erosion of PLGA by hydrolytic degradation is the crucial factor controlling the release of RAPA, other multiple factors are actively involved including diffusion of RAPA from PLGA and swelling of PLGA. The release of DOX from np-Au along the PLGA matrix can also contribute to the release of RAPA.

A key point is that the use of an implant is intended to provide localized therapy where the drug diffuses out of the implant and the localized concentration in and near the tumor tissue is of key importance. It is actually desired to reduce the systemic distribution to minimize side effects that would occur if DOX was injected intravenously. The therapeutic dose of DOX depends on many factors including the nature and the size of the tumor. The recommended dosage of DOX by the U.S. FDA is 60–75 mg m^−2^ or 1.2–2.4 mg kg^−1^, as an intravenous injection administered at 21 days interval. However, how much of this drug reaches tumor sites in humans is still a subject of research. In a mouse model, 1 ng DOX mg^−1^ of tumor has been reported for DOX injected without any carrier and up to 4–25 ng DOX mg^−1^ for a tumor with liposomes when 5 mg kg^−1^ of DOX was administered [73,74]. Although this depends on various factors, DOX in the ng range is sufficient to suppress tumor growth [73]. Therefore, the loading amount of 100 µg per np-Au millirod in our study is significant because (1) DOX releases locally, and could directly attack tumor cells with limited systemic side effects, and (2) sustained release of DOX causes accumulation improving effectiveness. Since the mode of delivery is quite different between localized delivery and systemic delivery, the dose cannot be directly compared. Additionally, the dimensions and the number of millirods could be varied to adjust the dose.

Implants inside the body can easily encounter the circulatory system creating unexpected problems. Therefore, it is imperative to understand the role of serum proteins on the drug release mechanism due to corona formation. We used 10% newborn calf serum as a medium for drug release to mimic the more realistic conditions. Figure 11D shows the release of a low and steady dose of DOX from both np-Au and np-Au@PLGA/RAPA for 25 days. In the first 24 h, the DOX released is negligible suggesting that protein corona forms instantly around the implants regardless of PLGA coating. After 24 h, we observed burst release suggesting a sudden escape of DOX from a protein barrier by diffusion for 48 h, followed by sustained release. Since DOX is gradually releasing from np-Au@PLGA/RAPA through the PLGA matrix, we can assume that PLGA is degrading, and hence RAPA is also releasing steadily. However, due to the extreme background signals from proteins in serum, RAPA’s release profile could not be experimentally verified using UV–Vis.

### 3.8. Drug Release Kinetics

To better understand the release mechanism of DOX from np-Au and np-Au@PLGA/RAPA and that of RAPA from np-Au@PLGA/RAPA, the drugs release profiles were fitted with different commonly used mathematical models for drug release. The best models were chosen based on R^2^ values and kinetic data are presented in Table 1. The Korsmeyer−Peppas model best fits the release of DOX from both np-Au and np-Au@PLGA/RAPA and RAPA from np-Au@PLGA/RAPA in the buffer at all pH. The initial 60% of the drug release data were fitted to a semiempirical Korsmeyer–Peppas equation [75]

$$\frac{M_{t}}{M_{\infty }}{\;=\;k\;\times \;t}^{n}$$

where *M*_t_/*M*_∞_ is the drug release fraction at time t, *k* is the release rate constant, and n is the release exponent that indicates the drug release mechanism.

For a non-swellable cylindrical sample, *n* = 4.5 corresponds to the Fickian diffusion, 4.5 < *n* < 1 corresponds to the anomalous (non-Fickian) transport, *n* = 1 corresponds to Case II (zero-order release), and *n* > 1 corresponds to super Case-II diffusion [76,77]. According to this classification, DOX released from np-Au at pH 5.5 (*n* = 0.47) and 6.6 (*n* = 0.98) was controlled by anomalous transport. Interestingly, at a pH 5.5 value of *n* is so close to 0.45 that it can be considered Fickian diffusion and at pH 6.6 value of *n* is so close to 1.00 that it can be considered a zero-order release. At pH 7.4 (*n* = 1.14), the release of DOX from np-Au is controlled by super Case-II diffusion. The DOX released from np-Au@PLGA/RAPA at pH 5.5 (*n* = 1.08) and 6.6 (*n* = 1.13) is controlled by super Case-II diffusion whereas at pH 7.4 (*n* = 0.84) it is controlled by anomalous transport. The value of *n* obtained for RAPA released from np-Au@PLGA/RAPA at pH 5.5, 6.6, and 7.4 were 1.19, 0.69, and 0.32, respectively. This proves that pH had a significant impact on degrading PLGA film and hence in the drug release process. The release mechanism of DOX from np-Au@PLGA/RAPA is the super Case-II diffusion, anomalous transport, and Fickian diffusion at pH 5.5, 6.6, and 7.4, respectively. Unlike in buffers, the release of DOX from both np-Au and np-Au@PLGA/RAPA in NBCS better fit the linear polynomial equation to support the zero-order release model. This underscores the necessity of determining the release kinetics of drugs in a medium similar to the real environment rather than just in the buffer solution, as the release kinetics may vary substantially with the nature of the medium.

## 4. Conclusions

We demonstrated that np-Au millirods possess outstanding DOX loading capacity without any chemical modification of DOX or np-Au. We discussed the possible mechanisms of loading of DOX onto np-Au, which involve Au–π and Au–carbonyl interactions between Au and DOX and dimerization of DOX through π–π stacking. We assumed that sustained release of DOX from np-Au occurs only due to the breaking of π–π stacking under the experimental conditions (37 °C, pH 5.5–7.4), leaving the rest of the DOX inside np-Au. Nearly 100% of the remaining DOX can be removed by dipping the np-Au on DMSO for 1 h. Additionally, we identified the DOX-loaded np-Au@PLGA/RAPA as a promising candidate for sustained co-release of RAPA and DOX, which may substantially avoid dosage related toxicity. During co-release, the RAPA released from PLGA may weaken the drug resistance capacity of tumor cells and therefore, the monomeric DOX released from np-Au has a higher possibility to enter cancer cells. The advantage of this macroscopic-sized nanostructured implant is that its dimensions can be easily varied, and hence the dosage can possibly be delivered according to the patient’s needs. Implants made from nanoporous gold also have the possibility for external stimulation of drug release by illumination with an infrared laser or application of ultrasound. The size of the implant was chosen thinking that it can be placed inside or near the tumor volume using an implant applicator or image-guided catheter in a minimally invasive manner [78]. Additionally, np-Au can generate localized surface plasmon resonance [79], a property that can be used for heating the surrounding of the nanostructures using a near-infrared laser [80]. This property can be applied for increasing the release rate of drugs or directly heat-killing cancer cells. Therefore, we need to understand the drug loading and release behavior of np-Au for future development and improvement of the drug delivery system. The tunability of pore size accessible by varying the dealloying conditions and also the possibility for surface modification can provide means of varying the release profile using implants of the same dimensions.

## Figures and Tables

**Table 1:** Kinetic parameters for DOX/RAPA release from np-Au-based implants.

Model	Release Medium	Drug	Implant	R^2^	*n*	k
Korsmeyer-Peppas	pH 5.5	DOX	np-Au	0.942	0.47	1.66
		DOX	np-Au@PLGA/RAPA	0.983	1.08	0.10
		RAPA	np-Au@PLGA/RAPA	0.984	1.19	0.22
	pH 6.6	DOX	np-Au	0.997	0.98	0.28
		DOX	np-Au@PLGA/RAPA	0.988	1.13	0.07
		RAPA	np-Au@PLGA/RAPA	0.991	0.69	2.28
	pH 7.4	DOX	np-Au	0.948	1.14	0.19
		DOX	np-Au@PLGA/RAPA	0.993	0.84	0.27
		RAPA	np-Au@PLGA/RAPA	0.994	0.32	11.09
Zero order	pH 7.4 (NBCS)	DOX	np-Au	0.991	NA	0.04
		DOX	np-Au@PLGA/RAPA	0.997	NA	0.03

## Data Availability

The data presented in this study are available within the article and its Supplementary Materials.

## Supplementary Materials

The following are available online at https://www.mdpi.com/2079-4991/11/1/208/s1, Figure S1: UV-Vis absorption spectra of DOX and RAPA, Figure S2: standard calibration plots, Figure S3: photographic image showing the importance of NaCl to prevent degradation of DOX on np-Au surface.
