# Supplementary material for: Nanoporous Gold Monolith for High Loading of Unmodified Doxorubicin and Sustained Co-Release of Doxorubicin-Rapamycin
Source: Nanomaterials (Basel). 2021 Jan 15;11(1):208. doi: 10.3390/nano11010208 (PMC7830488; doi:10.3390/nano11010208)
Supplement: Supplementary file 1 [file nanomaterials-11-00208-s001.pdf]

nanomaterials

Supporting Information

Nanoporous Gold Monolith for High Loading of Unmodified Doxorubicin and Sustained Co-Release of Doxorubicin-Rapamycin

Jay K. Bhattarai, Dharmendra Neupane, Bishal Nepal, Alexei V. Demchenko and Keith J. Stine *

Department of Chemistry and Biochemistry, University of Missouri–St. Louis, Saint Louis, MO 63121, USA; jkbxv3@mail.umsl.edu (J.K.B.); dnfzf@mail.umsl.edu (D.N.); bnff8@mail.umsl.edu (B.N.); demchenkoa@umsl.edu (A.V.D.)

* Correspondence: kstine@umsl.edu

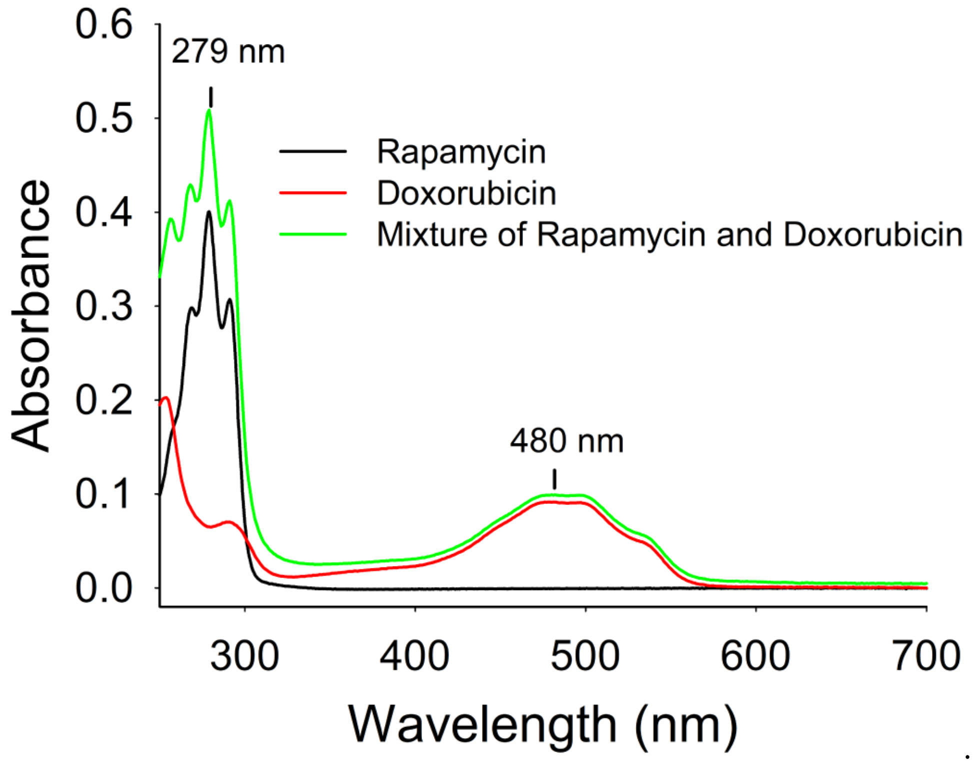

Figure S1. UV-Vis absorption spectra of 10 mM rapamycin (RAPA) and doxorubicin (DOX) separately and as a mixture. Peak wavelength of 480 nm and 279 nm were used for quantifying the release of DOX and RAPA, respectively.

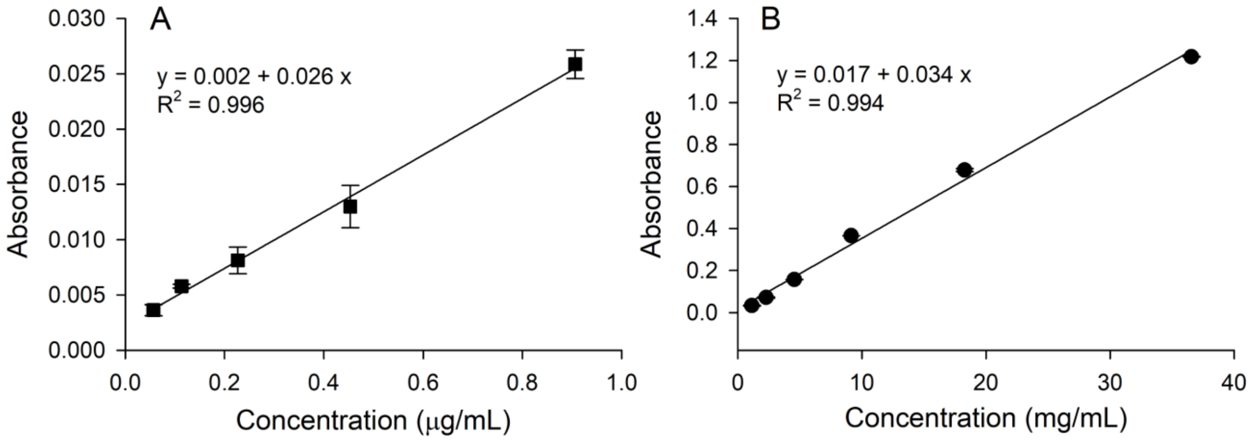

Figure S2. Standard calibration plots of (**A**) DOX and (**B**) RAPA.

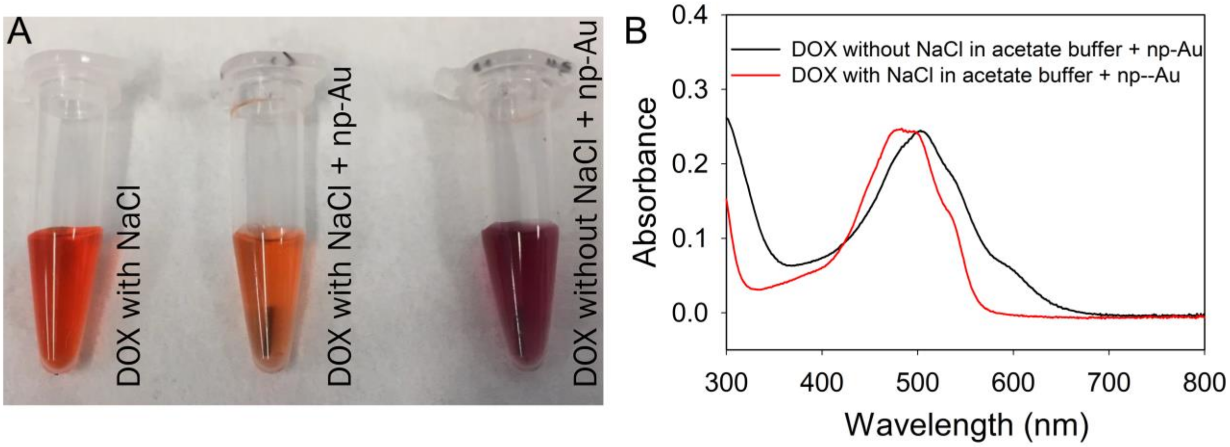

Figure S3. Photographic image and UV-spectra of DOX after 24 h of loading onto np-Au at room temperature showing importance of NaCl to avoid degradation in the presence of np-Au.
